# Current Status and Management Strategies of Obstetric Hemorrhage Using Contrast-enhanced Dynamic Computed Tomography: Correspondence

**DOI:** 10.31662/jmaj.2025-0072

**Published:** 2025-05-30

**Authors:** Hinpetch Daungsupawong, Viroj Wiwanitkit

**Affiliations:** 1 Private Academic Consultant, Phonhong, Lao People’s Democratic Republic; 2 Department of Research Analytics, Saveetha Dental College and Hospitals, Saveetha Institute of Medical and Technical Sciences, Saveetha University, Chennai, India

**Keywords:** obstetrics, diagnosis, hemorrhage

Dear Editor,

We would like to share ideas on the publication “Current Status and Management Strategies of Obstetric Hemorrhage Using Contrast-enhanced Dynamic Computed Tomography in a Representative Tertiary Perinatal Medical Center in Japan ^(1)^.” This retrospective study offers critical information on the clinical outcome of intrauterine hemorrhage. Treatment and diagnostic procedures are given special attention, such as contrast-enhanced dynamic computed tomography (CE-dCT). A primary merit of the study is the use of CE-dCT in 85% of cases, which is a new method that may increase diagnostic accuracy. However, the retrospective methods introduce considerable confounding variables, particularly in the identification of contrast leakage in arteries. It is unclear whether all patients were treated using the same diagnostic technique, which could influence the results. Using prior data without a control group makes it difficult to directly correlate specific outcomes to interventions and diagnostic approaches. In the future, a direct comparison of treatment effects based on various diagnostic procedures and management strategies could address this issue.

One critical aspect that requires greater explanation is the treatment decision-making process, particularly when choosing between conservative and invasive treatments such as transcatheter arterial occlusion. The study discovered that bleeding could be stopped in 57% of patients with conservative treatment. Although 43% of patients required invasive treatment, the factors that influenced the choice to transition from conservative to invasive treatment were not addressed. Patient-specific characteristics, such as bleeding intensity, comorbidities, and clinical response to early treatment, were not well explored. Understanding these aspects may be critical for customizing management protocols. This study may potentially provide more insight into the impact of various therapies. Long-term concerns include recovery time for patient outcomes and maternal disease.

Although the statistical analysis in this study is valuable, a more specific breakdown of the causes of bleeding and intervention results, such as the need for blood transfusions, would be advantageous. Rates of complications and patient survival could be stratified by the etiology of bleeding. This would provide a better understanding of the elements driving results. According to studies, blood loss and transfusion quantities are similar, independent of the cause of intrauterine bleeding. However, it is uncertain whether these averages conceal significant differences between groups that may be relevant in the study analysis. Multivariate regression models were used to account for confounding variables such as comorbidities, gestational age, and previous parity. This could help to improve conclusions and produce more robust outcomes.

Finally, one potential option for future study is to assess the cost-effectiveness and feasibility of using CE-dCT as a routine diagnostic tool for intrauterine hemorrhage. Although this work reveals the high leakage detection rate CE-dCT, the therapeutic value of the technique, particularly its impact on treatment results and patient recovery, requires further evaluation. Furthermore, we will broaden the scope of our research to investigate new treatment modalities, such as the function of blood-saving procedures and personalized medicine approaches, which may aid our understanding of ways to improve patient care in the management of uterine hemorrhage. Future research should focus on the long-term results in patients who underwent various treatment modalities, and which techniques best balance safety, efficacy, and cost-effectiveness.

## Article Information

### Conflicts of Interest

None

### Author Contributions

Hinpetch Daungsupawong and Viroj Wiwanitkit contributed equally to the ideas in the study. Hinpetch Daungsupawong was responsible for writing and analysis. Viroj Wiwanitkit was responsible for supervision. Both authors approved the manuscript.

### Data Availability

No new data were generated.

### AI Declaration

The authors used a language editing computational tool in preparation of the article.
